# Data dreams: planning for the future of historical medical documents

**DOI:** 10.5195/jmla.2018.444

**Published:** 2018-10-01

**Authors:** Lorraine Dong, Polina Ilieva, Aimee Medeiros

**Affiliations:** Project Manager, National Network of Libraries of Medicine Evaluation Office, Health Sciences Library, University of Washington; Archivist, UCSF Archives and Special Collections, University of California, San Francisco; Program Director and Assistant Professor, History of Health Sciences, University of California, San Francisco

## Abstract

Historical medical collections with privacy-sensitive information are a potentially rich source of social, behavioral, and economic data for a wide array of researchers. They remain relatively undiscoverable and at risk for destruction, however, because of their restricted content and challenging media formats. Team members from two institutions—the University of California, San Francisco, and the University of Texas at Austin—present their respective initiatives to create digital archives and databases that address the privacy and technological challenges of such collections. In doing so, they also argue for the importance (and feasibility) of medical libraries and archives to take the initiative to preserve and make accessible historical patient data.

At the Archivists and Librarians for the History of Health Sciences annual meeting in Nashville in May 2017, a panel of archivists and researchers discussed ongoing efforts from across the country to make privacy-restricted archival medical records more readily available as research data. The session, titled “Preserving and Providing Access to Historical Patient Data,” brought together presenters from the University of California, San Francisco (UCSF); the University of Texas at Austin (UT); and Johns Hopkins University (JHU) who had a common interest in protecting historical, paper-based medical records in order to make them discoverable and accessible through digital means.

The three institutions are working on projects to preserve and provide access to collections of medical records that contain patient information. The UCSF and JHU projects are efforts being conducted in the universities’ archives and special collections departments, and the UT project is based in the School of Information and funded by a 3-year, $763,000 grant (#11500653) from the Andrew W. Mellon Foundation. While the projects are not connected by their collections, they all share a focus on medical record collections that have high research value potential but remain relatively unavailable. These projects also address similar privacy and technological challenges, namely creating digital archives and databases that allow users to discover privacy-sensitive handwritten records while still adhering to medical information privacy laws.

In this History Matters column, team members from the UCSF and UT projects discuss why the preservation of historical medical records, including those with information currently restricted by federal and state privacy laws, is vital for longitudinal studies and other research requiring historical data. Furthermore, the authors deliberate on the strides that they have made in their own projects to show how privacy-sensitive collections can transition into becoming more accessible and easily consumable primary source data. The means to providing access differ for these two institutions, indicating the multiple routes that medical libraries and other custodians can take for their own historical medical records.

## RECOGNIZING VALUE IN HISTORICAL MEDICAL RECORDS

The long-term research value of historical medical records is not readily apparent to everyone in the medical and information fields. Why save records that most people cannot view for decades and are costly to securely store and provide access to? According to federal Health Insurance Portability and Accountability Act (HIPAA) privacy laws, medical records with personal health information that are maintained in US-covered entities must be closed for research for fifty years from the death of the individual [1]. For medical records in state repositories that are not covered by federal privacy laws and in covered entities in states with more stringent health information privacy laws, the restrictions are often longer. Closer examination of several archival medical record collections, however, reveals that such records contain data with enormous potential for multiple types of medical and social science research.

From the start of UT’s Central State Hospital Digital Library and Archives (CSH) project, the distinctive background of the hospital and the comprehensiveness of its archives made the institution’s archival collection an ideal candidate for a proof-of-concept project to demonstrate that health sciences collections with restricted information could be made into accessible sources of data that would be of high value to medical and academic scholars. The Petersburg, Virginia, hospital was founded at the end of the Civil War to become the first asylum anywhere in the United States to exclusively serve the African American population. The historical records of CSH, which date as far back as the 1860s, include individual patient files, admission registers, and board meeting minutes that contain medical and other personal information about patients.

A preliminary analysis of CSH admission register data suggests that the records are a rich source of data on historical African American mental health care, a little studied area in the history of medicine and in contemporary medical studies. At present, there are information disparities in regard to understanding mental health and mental health care among African Americans, due in part to a systemic racial bias regarding the health care of minority communities and a lack of access to inactive medical records that focus on these populations.

**Figure 1:**
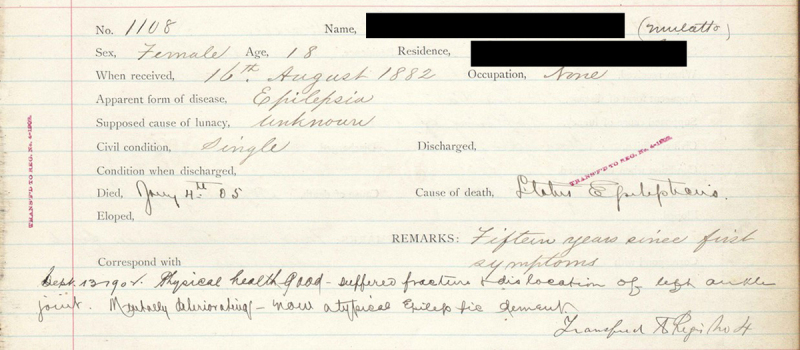
Central State Hospital Digital Library and Archives (CSH) record for an individual admitted in 1882 for “epilepsia”

The analysis shows that from 1870 to 1941, nearly 20,000 African Americans were admitted to CSH. For each person admitted to the hospital, the registers included a dozen categories of demographic data, for example, type of admission, diagnosis, and cause of illness (Figure 1). In the first 4 decades of the hospital’s history, 220 different diagnoses were assigned to approximately 5,000 patients. Two members of the CSH project team examined these diagnoses and translated them into contemporary diagnoses from the *Diagnostic and Statistical Manual of Mental Disorders, 5th edition.* As a result, they were able to group the early diagnoses into 8 modern categories: dementia, alcoholism, psychotic disorders, affective disorders, developmental disorders, epilepsy, and “unknown.” In today’s psychiatric classification system, 66% of the early diagnoses would be categorized as psychotic disorders.

An equally compelling collection of historical patient data is found at UCSF. Currently, UCSF’s Special Archives and Department of Anthropology, History and Social Medicine are spearheading a project to digitize data from 7 million paper-based patient records documenting health care services provided at UCSF’s health care facilities stretching back into the 19th century. Collectively, these records measure approximately 82,000 linear feet, or 102,000 boxes. They feature point-of-care data from the University Hospital’s Medical Clinic, Pediatric Clinic, Obstetrical-Gynecological Clinic, Surgery Clinic, and Mt. Zion Medical Center.

The records capture vital information, including demographics, diagnoses, procedures, laboratory tests, vital statuses, medications, nurses’ notes, psychological observations, physicians’ drawings and notes, documentation from social welfare reports, and patient-physician interactions. These data allow innovative and complex research in medicine, public health, social and population sciences, and medical humanities. For example, public health scholars will be able to research data that provide a unique opportunity to understand how Americans and health care facilities previously responded to huge changes in payments and the provider landscape. Historians would be able to examine the interpersonal exchanges that have contributed to long-term trends in health care disparities. Scholars engaged in medical humanities will be able to analyze these data using social network analysis science in order to assess the impact that health care has had on the evolution of medicine in the United States since its scientific revolution in the late nineteenth century. There is also the possibility to link singular personal and family histories to larger sociological histories to promote health and the alleviation of suffering today.

**Figure 2:**
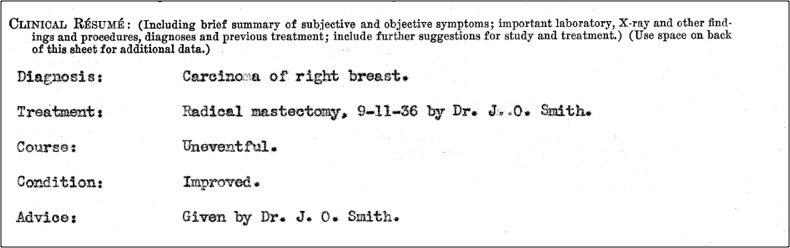
Breast cancer treatment description in a “Clinical Resume”

The UCSF paper-based patient record set offers a wealth of historical patient data. While each record documents an individual experience, these data also speak to larger historical trends in health care, making them have both qualitative and quantitative value. In one of the patient records collected by UCSF, a woman’s treatment for breast cancer was recorded in a section titled “Clinical Resume” (Figure 2). This entry not only documents care that this one individual received, but also illustrates how radical mastectomy was the first line of treatment in cases of breast cancer during the first decades of the twentieth century [2]. The personal account featured on the patient record documents the patient’s positive attitude and the physician’s dedication to treat the woman. At the same time, these data points about procedures taken and strategies adopted are equally valuable in understanding larger trends in health and health care delivery.

**Figure 3:**
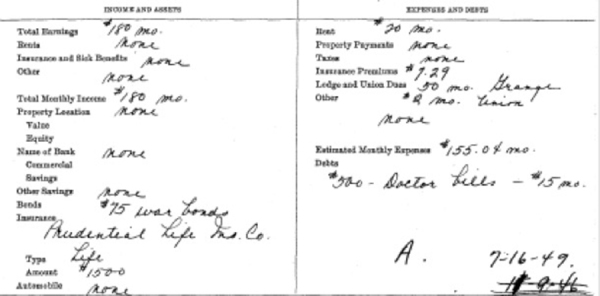
Economic data from an admission record

While historical health information is extremely significant and has the potential to directly inform medical, social, behavioral, and economic sciences research, non-health-specific data that carry their own inherent value are also featured on these paper-based records and extend the significance of these records. For instance, records documenting the routine procedure of patient intake often include socioeconomic data (Figure 3). Admissions records for many decades contained sections on “income and assets” and “expenses and debts.” Each one features data meaningful to researchers from diverse fields, including the distribution of war bonds, car ownership, and the cost of living. Scientists would also value the demographic data (e.g., race, sex, marital status, nationality, and age) recorded on these forms.

From iron lungs to genetic testing, technologies have transformed patient care and health outcomes. Historical patient data demonstrate the realities of implementing technologies and how they have or have not revolutionized medicine and the health sciences.

The successful completion of the UCSF project, which will result in the unprecedented access to, and malleable state of historical patient data will facilitate research to be conducted on pertinent issues, such as health inequalities, the impact of new technologies in the health services, population health, and the functioning of health care systems.

## MAKING HISTORICAL MEDICAL COLLECTION AVAILABLE

At present, the primary users of extant historical medical records are descendants exploring their family histories and professional genealogists. Medical researchers and others in search of aggregate historical data first face the difficulty of gaining permission to access substantial sets of paper-based records with often restricted health information. Then, they face the challenge of aggregating the data by hand. Digitization only solves part of this research challenge by ostensibly providing digital preservation of the historical documents. Discoverability of medical record collections and specific documents is made possible through online finding aids, databases, and catalogs. Searching for specific words and phrases, without compromising medical privacy laws, remains on the frontier of health sciences informatics research. The potential for historical medical records to become searchable and manageable data that can be used for large-scale and longitudinal studies remains a tantalizing goal for several groups, including the UT and UCSF teams.

**Figure 4:**
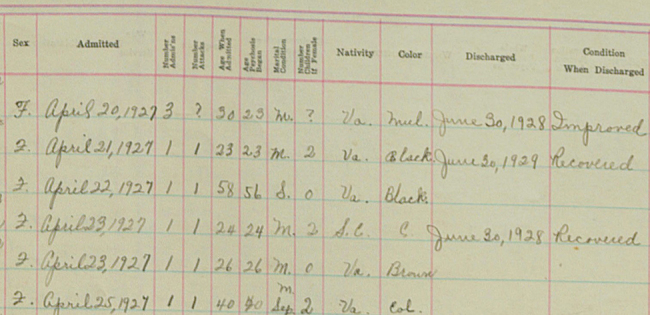
Example of handwriting from a 1920s CSH patient register

The CSH project aims to make the hospital’s digitized historical records more readily discoverable and, over time, accessible to a wide variety of users, including CSH staff, patients’ family members, researchers, and the general public. More generally, the project team is developing workflows and technologies that they intend to make freely available to any institution or organization that has collections with sensitive information and wishes to make them more available through digital means. At present, the team is working on strategies to create pertinent metadata for handwritten records and to crowdsource transcriptions so that researchers can search and sort the records at a highly granular level (Figure 4). These steps will also allow differing levels of access to the records, depending on the type of information requested and the credentials of the information seeker.

UCSF’s efforts are following along similar lines. As funding is made available, the team is digitizing the records, conducting optical character recognition, and capturing metadata. Beginning in 2016, historians and archivists from several institutions (Johns Hopkins University, Harvard University, Western Michigan University, the Wellcome Trust, Yale University, and the New York Academy of Medicine Library) came together to brainstorm about the possibility of developing a scalable database and discovery tools that will allow unlocking the potential of historical patient data and making possible breakthrough research, while providing security and assurance of ethical use of these records. The Historical Patient Data Portal (HPDP) is currently in the conceptual phase.

By enhancing data utility, the HPDP will allow scientists to address significant research areas including health inequalities, the impact of new technologies in health services, population health, and the functioning of health care systems. Until now, researching historical patient data has been plagued by several obstacles, including the fact that the data are bound to paper records and, if digitized, are stored on disparate databases. Without their digitization, these data are not necessarily considered usable, relational, or amenable to data research. If digitized, their diverse locations compromise computational analysis. The modernization of historical patient datasets is imperative, and the HPDP will prove the usefulness of this process as it ushers in a new era in social, behavioral, and economic health research, which is not bound by the original medium of the collected data.

HPDP’s intellectual merit is its capacity to transform historical patient data research by providing unprecedented access to this type of information and the means for its analysis in a secure environment. HPDP will help researchers overcome barriers, real and imagined, by streamlining discovery and use of historical patient datasets. There will be increased awareness of the datasets once they are digitized and available for analysis through the HPDP. Subsequently, this project promotes historical data preservation by ensuring the data’s usability in a secure environment.

## CONTINUING ADVOCACY

As imminent advancements in technologies and techniques are anticipated to make archival materials with sensitive or legally restricted information more readily discoverable and accessible, it is more important than ever for medical researchers, historians, and information professionals to champion the preservation of these underutilized collections. Two major reasons for why custodians of inactive medical records do not retain them for future archival use—the cost of maintaining inactive health records and the current inability to accurately and quickly redact restricted information—continue to put these collections at risk for destruction.

Federal and state medical information privacy laws will always impose some level of restriction on what types of information can be seen, by whom, and when, although they will continue to change over time to accommodate shifting societal attitudes toward medical record privacy and rights to information access. It is, therefore, vital for aggregation and anonymization research to push forward and go beyond current endeavors.

In the meantime, one way that librarians, archivists, record managers, and traditional “end users” of collections, including historians, can be advocates for the long-term preservation of historical medical record collections is to become more familiar with the hidden and less accessible collections in their own repositories that are restricted for legal and ethical reasons. They can investigate the research potential of these collections, especially in terms of the records’ possible contributions to longitudinal studies and researchers’ demand for such data. Whether anecdotal observations or formal studies, these reports will serve as much-needed evidence to institutional administrators, funders, and colleagues alike of the enduring value of historical medical records.
